# Experimental studies on the effects of bolt parameters on the bearing characteristics of reinforced rock

**DOI:** 10.1186/s40064-016-2580-z

**Published:** 2016-06-24

**Authors:** Liang Cheng, Yidong Zhang, Ming Ji, Kai Zhang, Minglei Zhang

**Affiliations:** Key Laboratory of Deep Coal Resource Mining, Ministry of Education of China, School of Mines, China University of Mining and Technology, Xuzhou, 221116 China; State Key Laboratory of Coal Resources and Safe Mining, China University of Mining and Technology, Xuzhou, Jiangsu China

**Keywords:** Bolt, Surrounding rock, Roadway, Reinforced rock, Stress, Displacement

## Abstract

Roadways supported by bolts contain support structures that are built into the rock surrounding the roadway, referred to as reinforced rocks in this paper. Using physical model simulation, the paper investigates the bearing characteristics of the reinforced rock under different bolt parameters with incrementally increased load. The experimental results show that the stress at the measurement point inside the structure varies with the kinetic pressure. The stress increases slowly as the load is initially applied, displays accelerated growth in the middle of the loading application, and decreases or remains constant in the later stage of the loading application. The change in displacement of the surrounding rock exhibits the following characteristics: a slow increase when the load is first applied, accelerated growth in the middle stage, and violent growth in the later stage. There is a good correlation between the change in the measured stress and the change in the surrounding rock displacement. Increasing the density of the bolt support and the length and diameter of the bolt improves the load-bearing performance of the reinforced rock, including its strength, internal peak stress, and residual stress. Bolting improves the internal structure of the surrounding rocks, and the deterioration of the surrounding rock decreases with the distance between the bolt supports.

## Background

Since the time bolting was determined to be an effective method for securing rocks surrounding a roadway, a number of studies examined the performance of rock bolts both in the laboratory and in the field (Aydan 1989; Farmer 1975; Peng and Tang 1984; Stille et al. 1989; Sun 1984; Kang et al. 2015; Zhang et al. 2015). Analytical studies were also conducted to examine the influence of relevant parameters and to understand the interaction between the rock and the bolt (Dight 1982; Indraratna and Kaiser 1990; Holmberg 1991; Li and Stillborg 1999; Bobet 2002; Cai et al. 2004; Carranza-Torres 2009; Bobet and Einstein 2011). And there have been many investigations on bolting mechanisms, ranging from the initial suspension theory to the subsequent composite beam theory, compound arch theory, strength hardening theory and maximum horizontal principal stress theory (Hou and Gou 2000; Mark et al. 2007), and loosing-circle theory of surrounding rocks (Dong et al. 1994). The conclusion that bolting improves the load-bearing capability of the surrounding rock has been stated and confirmed in many studies. For roadways supported by bolts in the rock, different support structures for the surrounding rock have been suggested: e.g., the surrounding rock bearing circle proposed by Kang (1996, 1997), the internal and external bearing circle proposed by Li (2008), the support structure inside the roadway’s coal roof proposed by Zhang and Li (1999), the compound arch bearing structure by Yu et al. (2010), and reinforced rock by Song and Mu (1997), among others (Wu and Chai 1997; Zhu et al. 2000; Zhang et al. 2015; Cheng et al. 2015).However, few studies have investigated the load-bearing characteristics of a structure formed from both bolts and the surrounding rocks and the effect of the structure on the overall load-bearing capacity of the surrounding rocks. There are even fewer studies considering the effect of the bolt parameters on the load-bearing characteristics of such a structure.

The equilibrium stress inside a roadway changes after excavation. The stress in the shallow surrounding rock transforms from a triaxial stress state to a biaxial one, and the surrounding rock often contains many fracture zones. To maintain road stability, rock bolts can be used to support the roof and the two sides of the roadway. The pre-tightening force is applied overtime. With many bolts of appropriate length and separation distance, the bolt groups work together to create a load-bearing structure with a certain degree of strength, even with fractured, loose, or soft rocks. This load-bearing structure is referred to as a “composite rock-bolt bearing structure” in this study Song and Mu (1997). Bolting improves the internal stress state of the surrounding rock in this compound structure and increases the cohesion and internal friction angle, thus preventing the spread of the elastic zone in the rock surrounding the roadway and significantly decreasing rock deformation.

Through simulation of a physical model, the present study investigates the effect of different bolt parameters (i.e., the length and diameter of the bolts and the distance between the bolts) and the effect bolt parameters have on the structure’s load-bearing characteristics when subjected to gradual increased load.

## Experimental methods and measurements

### Design of experiments

Most rock bolt roadways used in engineering are coal roadways; therefore, coal was used in the present study to simulate the surrounding rock. The coal mass density is 1.35 × 10^3^ kg/m^3^. The cross section of the actual roadway was rectangular with a width of 3600 mm and a height of 3000 mm. The actual burial depth was 400 m, the vertical stress of the surrounding rock was 10 MPa.

The size of the model was 600 × 500 × 100 mm. The cross section of the designed roadway was rectangular, with a width of 120 mm and a height of 100 mm. Therefore the geometrical similarity ratio between the model and its prototype was 1:30. The density of the model rock was 1.5 × 10^3^ kg/m^3^ and hence the geometrical similarity ratio between the model and its prototype was 1:27. The load was applied to the top of the model and the bottom and two sides were immobilized.

The model material is concrete which comprised of sand, cement, gypsum powder, and water. The relative mass ratio was 8:0.7:0.3:0.1 when constructing the model. The compressive strength, cohesion, and internal friction angle of the model material were 0.97, 0.07, and 40 MPa respectively by averaging the test values, which were tested in China University of Mining and Technology. The test method followed Chinese standard GB/T23561. The test equipment was SANS servo mechanical press. The mass ratio of the sample was the same as that of the model material and the sample size was 70 × 70 × 70 mm. In the compressive test three samples were tested. In the shear test nine samples were tested with three different shear angles of 30°, 45°, 60° and each test of one angel contained three samples.

The fuse wires were used to simulate the bolts in the model. Two types of the fuse wires were used in the experiments. The breaking force of the two types of fuse wires were 22 and 66 N respectively according to a pullout force test. The breaking forces of the bolt in the prototype were 534kN and 1604kN, respectively.

The experimental process was as follows: (1) Simulation model building and pre-burying bolts; (2)Twelve hours after the model was built, the channel steels were removed, dried for 24 h, and painted, and the line was drawn; (3) Polymethyl methacrylate (PMMA) panels were used to immobilize the model. A jack was used to apply a pressure of 0.1 MPa to the roof of the model for 12 h; (4) Unloading the pressure, roadway excavation, and installment of tray; (5) Loading: An increase of 0.05 MPa was applied every half hour by the jack until the roadway was damaged.

## Experimental observation

**Fig. 1:**
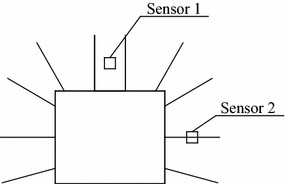
Layout of the measurement points

The layout of the stress sensors is shown in Fig. 1. Sensor 1 measures the roof stress of the reinforced rock, and sensor 2 measures the stresses on the side of the structure.

The displacement of the rock surrounding the roadway is the most straightforward and easy-to-measure variable that yields information on the load-bearing characteristics for roadways. In this study, the rock deformation was measured using measuring rulers.

### Experimental layout and kinetic pressure

A single-variable method was used for the test. The scheme and the maximum kinetic pressure coefficient for the model are shown in Table 1. The parameters in Table 1 are used with prototype parameters because it is familiar to the engineering staff.

**Table 1:** Experimental layout

Test number	Distance between bolts/mm	Distance between two rows/mm	Length/mm	Diameter/mm	Maximum kinetic pressure coefficient
1	No bolts				1.23
2	800	800	1800	18	2.42
3	600	800	1800	18	2.89
4	1000	800	1800	18	1.47
5	800	600	1800	18	2.89
6	800	1000	1800	18	1.90
7	800	800	2000	18	2.58
8	800	800	2400	18	2.70
9	800	800	1600	18	1.95
10	800	800	1600	30	2.80

Kinetic pressure means the applied load in the model. The kinetic pressure is variable but not dynamic. Kinetic pressure coefficient is equal to the applied load divided by the vertical stress. And the vertical stress is 10 MPa in the prototype and 0.37 MPa in the model.

It can be seen from Table 1 that increasing the density of the bolt support and the length and diameter of the bolts increases the maximum load coefficient for the surrounding rock.

**Fig. 2:**
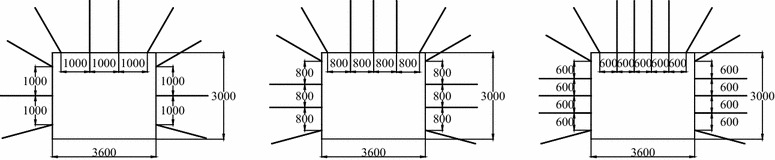
The location of rock bolts with different distance between bolts (prototype parameters)

The location of rock bolts with different distance between bolts is as shown in Fig. 2. The distance between bolts is used as prototype parameters because it is very similar to engineering staff.

**Fig. 3:**
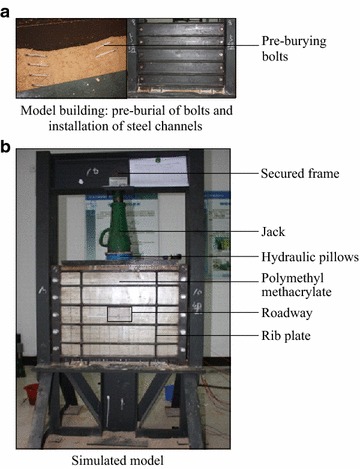
The experimental equipment

The experimental equipment is shown in Fig. 3.

A stress sensor is made of a polyvinyl chloride cube of 5 × 5×5 mm and two strain gauges (Ji et al. 2013). The rigidity of polyvinyl chloride cube is equal to that of model material. One strain gauge was stuck on the upper side of the polyvinyl chloride to monitor the vertical stress and the other one was stuck on the lateral side to monitor the horizontal stress.

## Discussion of experimental results

**Fig. 4:**
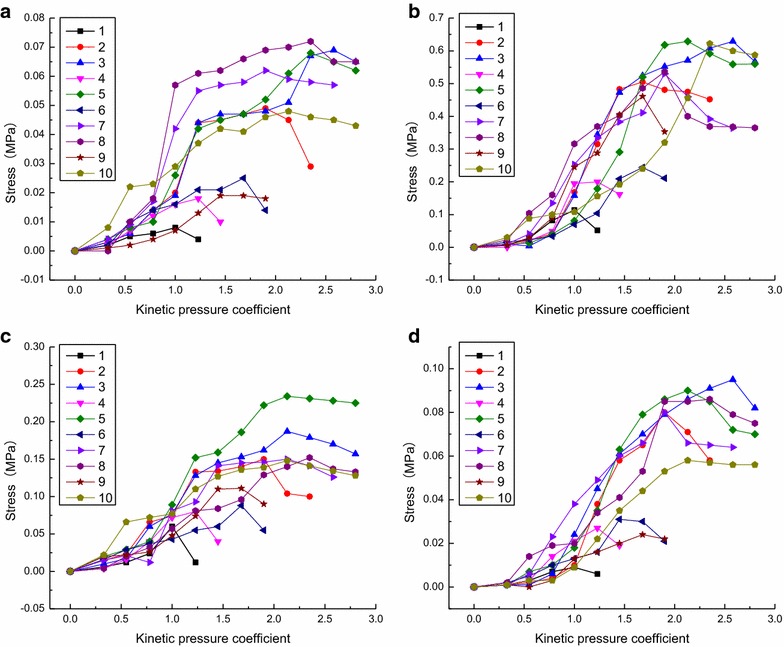
Relationship between stress and kinetic pressure coefficient. **a** Vertical stress at Sensor 1; **b** Vertical stress at Sensor 2; **c** Horizontal stress at Sensor 1; **d** Horizontal stress at Sensor 2

Once the data were obtained, the curves for the relationship between stress and the kinetic pressure coefficient for the measuring points of each group are plotted, as shown in Fig. 4.

 It can be seen from Fig. 4 that increasing the density of the bolt support and the length and diameter of the bolts increases the internal peak stress inside the reinforced rock. The characteristics of the variation of the measured stress with the kinetic pressure are also obtained: the stress increases slowly when the load is first applied, displays accelerated growth in the middle of the loading application, and decreases or remains constant in the later stage of the loading application.

**Fig. 5:**
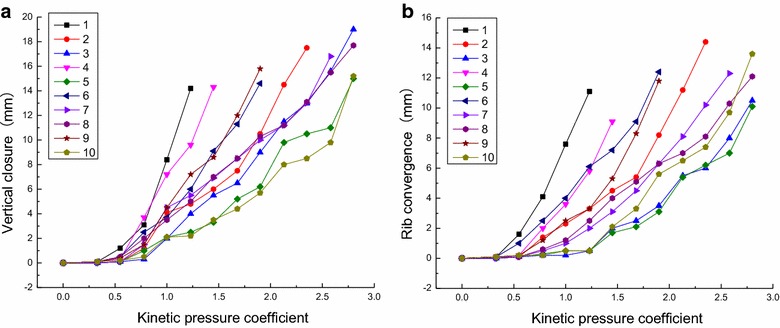
Relationship between the surrounding rock displacement and kinetic pressure. **a** Vertical closure; **b** Rib convergence

The curve for the relationship between the surrounding rock displacement and the kinetic pressure of each group is plotted in Fig. 5.

The following observations can be made from Fig. 5.

1. The displacement of the roadway roof and sides increases as the kinetic pressure increases. In real applications, if the rock around the roadway is affected by the kinetic pressure, the securing of the surrounding rock must be enhanced, including the reinforced support in the initial stage of the roadway and corresponding remedial measures for the kinetic pressure effects.
2. The variation of the surrounding rock displacement with the kinetic pressure exhibits the following characteristics: it increases slowly in the beginning, displays accelerated growth in the middle stage, and grows extremely quickly in the later stage. Increasing the density of the bolt support and the length and diameter of the bolts decreases roadway deformation.

**Fig. 6:**
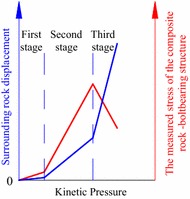
The variation trend of the measured stress and the surrounding rock displacement

The analysis of the data in Figs. 4 and 5 reveals a general variation trend that is divided into three stages, as shown in Fig. 6.

1. First stage

In this stage, the measured stress and the corresponding displacement of the surrounding rock grows slowly. During the initial loading, the measured stress around the roadway increases very slowly, and the surrounding rock displacement is very small. Regardless of the roadway support, the bolts showed little effect on both the stress and the surrounding rock displacement. This is because the surrounding rock in the first stage remains almost undamaged with a certain degree of load-bearing capacity. The bolt only weakly reinforces the surrounding rock. The kinetic pressure in this stage is 0–0.5σ_1_.

2. Second stage

In this stage, the measured stress and the corresponding displacement of the rock surrounding the reinforced rock exhibits much larger growth than the first stage. The peak values are reached with the increased kinetic pressure. As the kinetic pressure for the roadway increases above 0.5σ_1_, the surrounding rock displacement increases and the stress of the bolt increases. The stress state of the reinforced rock improves and the load-bearing capacity of the bolt-supported surrounding rock is evidently increased compared to the rock without bolt support. As such, the load-bearing performance of the rock surrounding the roadway is improved with bolt support.

Comparing the change in internal stress when the surrounding rock is and is not supported by bolts, the bearing capacity of the bolted surrounding rock shows considerable reinforcement. The maximum applied load increase of twofold to threefold is observed, indicating that a load-bearing structure is formed inside the surrounding rocks after the bolt support is installed, namely the reinforced rock, which is formed from the bolt and surrounding rock. The inherent properties of the surrounding rock, the excavation radius of the roadway, and the parameters of the bolt support (such as the diameter, length, material quality of the bolts, and the separation distance between the bolts) all affect the bearing performance of the reinforced rock.

3. Third stage

In this stage, the measured stress inside the reinforced rock declines after reaching the peak values. The structure is gradually damaged after the measured stress inside the reinforced rock reaches the peak value, at which point the stress decreases although the rate is slow. The stress remains constant near certain values, suggesting that the structure experiences a certain degree of damage but still retains some strength. However, the roadway deformation rate versus load in the third stage is much larger than that in the first/second stage.

## Discussion of the effect of bolt parameters on the characteristics of the reinforced rock

### The roadway deformation rate versus load

Because the effect of time is not taken into account in this study, an “roadway deformation rate versus load” is proposed in order to investigate the relationship between the displacement of the surrounding rock and the applied load. The formula is shown as *η* = Δ*U*/Δ*P*, in which *η* is the roadway deformation rate in a certain stage (mm/MP); Δ*U* is the surrounding rock displacement in a certain stages (mm); and Δ*P* is the difference in the applied pressure in a certain stage (MPa).

The roadway deformation rate versus load reflects the capability of the reinforced rock to resist deformation under load. It includes the accelerated displacement of roof to floor and the sides.

Because the bolts only slightly reinforce the reinforced rock in the first stage, the effect of the bolt parameters on the measured stress and the roadway deformation rate versus load was only investigated in the last two stages.

### The effect of the distance between the bolt rows on the characteristics of the reinforced rock

**Fig. 7:**
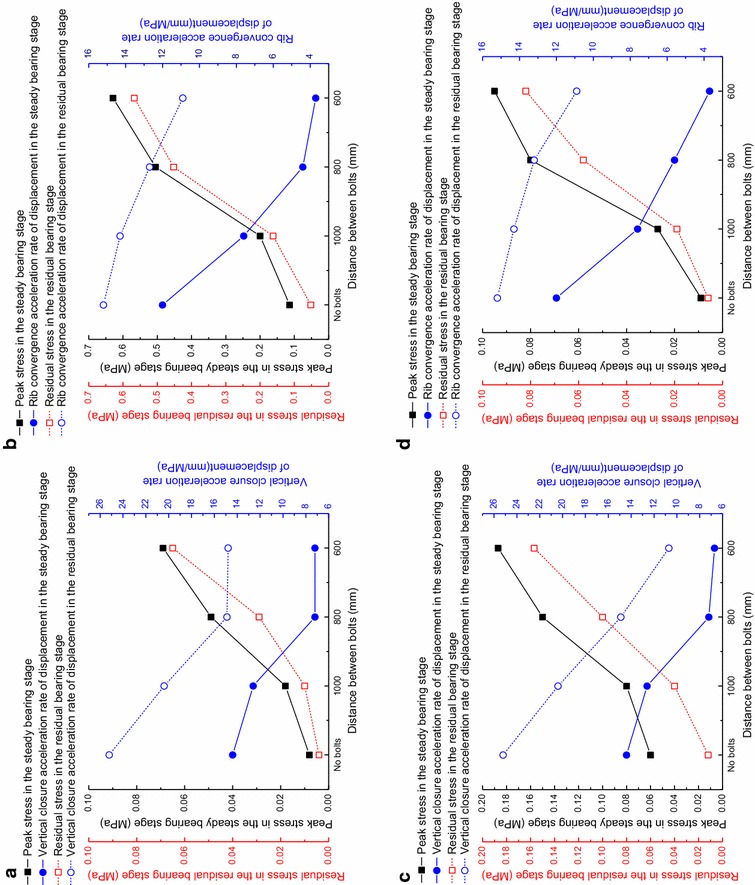
Load-bearing characteristics of the composite rock-bolt bearing structure with different distance between bolts. **a** Vertical stress at Sensor 1; **b** Vertical stress at Sensor 2; **c** Horizontal stress at Sensor 1; **d** Horizontal stress at Sensor 2

The measured stress inside the reinforced rock and the roadway deformation rate versus load at different distance between bolts are listed in Table 2. The bearing characteristics of the reinforced rock at different distance between bolts are obtained is shown in Fig. 7.

**Table 2:** The measured stress of the composite rock-bolt bearing structure and acceleration rate of surrounding rock displacement

Distance between bolts/mm	Second stage			Third stage			Percentage of stress decrease (%)
	Kinetic pressure coefficient range	Peak stress/MPa	Vertical closure acceleration rate of displacement/mm/MPa	Kinetic pressure coefficient range	Residual sress/MPa	Vertical closure acceleration rate of displacement/mm/MPa	
*(a) Vertical stress at Sensor 1*							
No bolts	0.50–1.00	0.008	14.4	1.00–1.23	0.004	25.2	50.0
1000	0.50–1.24	0.018	12.6	1.24–1.47	0.010	20.4	44.4
800	0.50–1.95	0.049	7.2	1.95–2.42	0.029	14.9	40.8
600	0.50–2.66	0.069	7.2	2.66– 2.89	0.065	14.8	5.8

Distance between bolts/mm	Second stage			Third stage			Percentage of stress decrease (%)
	Kinetic pressure coefficient range	Peak stress/MPa	Rib convergence closure acceleration rate of displacement/mm/MPa	Kinetic pressure coefficient range	Residual sress/MPa	Rib convergence closure acceleration rate of displacement/mm/MPa	
*(b) Vertical stress at Sensor 2*							
No bolts	0.50–1.00	0.114	12.0	1.00––1.23	0.052	15.2	54.4
1000	0.50–1.24	0.200	7.6	1.24–1.47	0.162	14.3	19.0
800	0.50–1.71	0.505	4.4	1.71–2.42	0.452	12.7	10.5
600	0.50–2.66	0.629	3.7	2.66–2.89	0.567	10.9	9.9

Distance between bolts/mm	Second stage			Third stage			Percentage of stress decrease (%)
	Kinetic pressure coefficient range	Peak stress/MPa	Vertical closure acceleration rate of displacement/mm/MPa	Kinetic pressure coefficient range	Residual sress/MPa	Vertical closure acceleration rate of displacement/mm/MPa	
*(c) Horizontal stress at Sensor 1*							
No bolts	0.50–1.00	0.060	14.4	1.00–1.23	0.012	25.2	80.0
1000	0.50–1.24	0.080	12.6	1.24–1.47	0.040	20.4	50.0
800	0.50–1.95	0.150	7.2	1.95–2.42	0.100	14.9	33.3
600	0.50–2.19	0.187	6.7	2.19–2.89	0.157	10.7	16.0

Distance between bolts/mm	Second stage			Third stage			Percentage of stress decrease (%)
	Kinetic pressure coefficient range	Peak stress/MPa	Rib convergence closure acceleration rate of displacement/mm/MPa	Kinetic pressure coefficient range	Residual sress/MPa	Rib convergence closure acceleration rate of displacement/mm/MPa	
*(d) Horizontal stress at Sensor 2*							
No bolts	0.50–1.00	0.009	12.0	1.00–1.23	0.006	15.2	33.3
1000	0.50–1.24	0.027	7.6	1.24–1.47	0.019	14.3	29.6
800	0.50–1.95	0.080	5.6	1.95–2.42	0.058	13.2	27.5
600	0.50–2.66	0.095	3.7	2.66–2.89	0.082	10.9	13.7

(1)The peak stress is the maximum stress in the second stage; (2) The residual stress is the minimum stress in the third stage; (3) The percentage of stress decrease is the percentage of decrease from the peak to residual stress

The following observations can be made from Fig. 7:

1. The data for the four models with different distance between bolts show that, under loading, the hoop stress of the rock surrounding the roadway is much larger and concentrated than the radial stress. Additionally, the side stress is more concentrated than the stress in the roof.
2. The load-bearing capacity of surrounding rock is increased with bolt support. Smaller distance between bolts produce larger kinetic pressures.
3. Decreasing the distance between bolts increases the reinforcing effect on the surrounding rock.
4. The residual load-bearing capacity of the surrounding rock after damage increases for the models with bolt support. The reinforcing effect is more pronounced with decreased distance between bolts.
5. Bolt support can strengthen the bearing capacity of the surrounding rock after the peak values are reached.
6. As the distance between bolts decreases, the roadway deformation rate versus load of the roof to floor also decreases. In other words, the ability to resist deformation increases with decreasing distance between bolts. After the peak stress is reached, the ability to resist deformation is lower than before the peak value was reached. The roadway deformation rate versus load at the sides display similar characteristics.

Because the distance between the rows of bolts had a similar effect on the load-bearing characteristics of the reinforced rock as the distance between bolts, it will not be discussed here.

### The effect of bolt length on the load-bearing characteristics of the reinforced rock

**Fig. 8:**
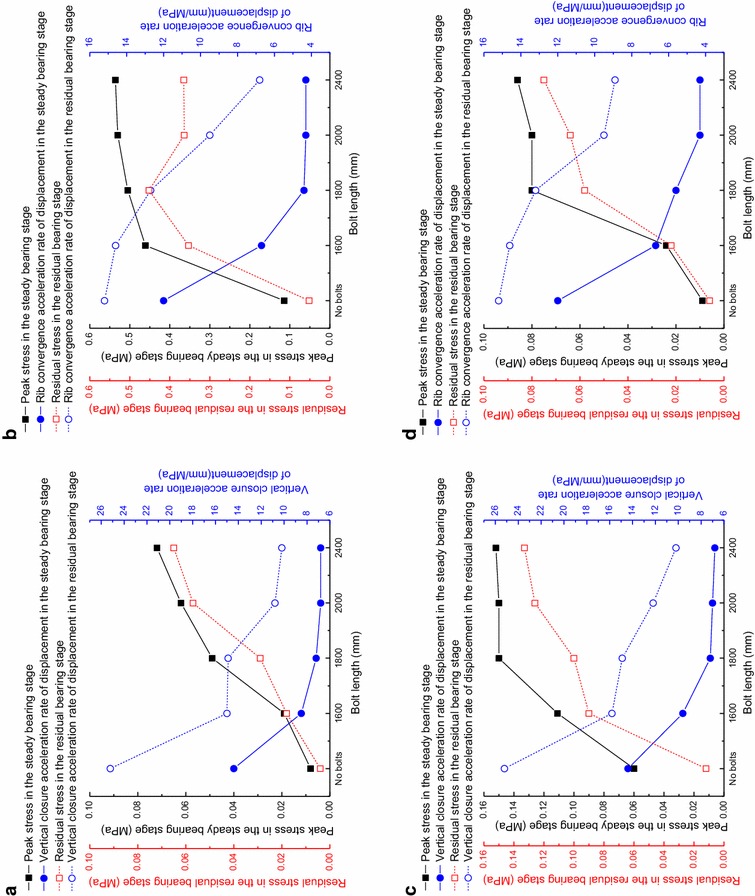
Load-bearing characteristics of the composite rock-bolt bearing structure with different bolt length. **a** Vertical stress at Sensor 1; **b** Vertical stress at Sensor 2; **c** Horizontal stress at Sensor 1; **d** Horizontal stress at Sensor 2

The measured stress inside the reinforced rock and the roadway deformation rate versus load for different bolt lengths are listed in Table 3. From Table 3, the load-bearing characteristics of the reinforced rock for different bolt lengths are obtained, as shown in Fig. 8.

**Table 3:** The measured stress of the composite rock-bolt bearing structure and acceleration rate of surrounding rock displacement

Bolt length/mm	Second stage			Third stage		
	Kinetic pressure coefficient range	Peak stress/MPa	Vertical closure acceleration rate of displacement/mm/MPa	Kinetic pressure coefficient range	Residual stress/MPa	Vertical closure acceleration rate of displacement/mm/MPa
*(a) Vertical stress at Sensor 1*						
No bolts	0.50–1.00	0.008	14.4	1.00–1.23	0.004	25.2
1600	0.50–1.47	0.019	8.5	1.47–1.95	0.018	15.0
1800	0.50–1.95	0.049	7.2	1.95–2.42	0.029	14.9
2000	0.50–1.95	0.062	6.8	1.95–2.58	0.057	10.8
2400	0.50–2.35	0.072	6.8	2.35–2.70	0.065	10.2

Bolt length/mm	Second stage			Third stage		
	Kinetic pressure coefficient range	Peak stress/MPa	Rib convergence closure acceleration rate of displacement/mm/MPa	Kinetic pressure coefficient range	Residual stress/MPa	Rib convergence closure acceleration rate of displacement/mm/MPa
*(b) Vertical stress at Sensor 2*						
No bolts	0.50–1.00	0.114	12.0	1.00–1.23	0.052	15.2
1600	0.50–1.71	0.461	6.7	1.71–1.95	0.353	14.6
1800	0.50–1.71	0.505	4.4	1.71–2.42	0.452	12.7
2000	0.50–1.95	0.530	4.3	1.95–2.58	0.364	9.5
2400	0.50–1.95	0.536	4.3	1.95–2.70	0.365	6.8

Bolt length/mm	Second stage			Third stage		
	Kinetic pressure coefficient range	Peak stress/MPa	Vertical closure acceleration rate of displacement/mm/MPa	Kinetic pressure coefficient range	Residual stress/MPa	Vertical closure acceleration rate of displacement/mm/MPa
*(c) Horizontal stress at Sensor 1*						
No bolts	0.50–1.00	0.060	14.4	1.00–1.23	0.012	25.2
1600	0.50–1.71	0.111	9.6	1.71–1.95	0.090	15.8
1800	0.50–1.95	0.150	7.2	1.95–2.42	0.100	14.9
2000	0.50–2.35	0.150	7.0	2.35–2.58	0.126	12.2
2400	0.50–2.35	0.152	6.8	2.35–2.70	0.133	10.2

Bolt length/mm	Second stage			Third stage		
	Kinetic pressure coefficient range	Peak stress/MPa	Rib convergence closure acceleration rate of displacement/mm/MPa	Kinetic pressure coefficient range	Residual stress/MPa	Rib convergence closure acceleration rate of displacement/mm/MPa
*(d) Horizontal stress at Sensor 2*						
No bolts	0.50–1.00	0.009	12.0	1.00–1.23	0.006	15.2
1600	0.50–1.71	0.024	6.7	1.71–1.95	0.022	14.6
1800	0.50–1.95	0.080	5.6	1.95–2.42	0.058	13.2
2000	0.50–1.95	0.080	4.3	1.95–2.58	0.064	9.5
2400	0.50–2.35	0.086	4.3	2.35–2.70	0.075	8.9

From Fig. 8, it can be seen that as the bolt length increases, the internal stress of the reinforced rock shows similar behavior as the decreased distance between bolts. However, some unusual behaviors also appear. As the bolt length increases, the maximum kinetic pressure coefficient also increases but the increasing rate of load decreases, as shown in Table 4.

**Table 4:** The maximum loading coefficient and acceleration loading at different bolt lengths

Bolt length/mm	Maximum loading coefficient	Accelerated loading coefficient
∞	1.23	–
1600	1.95	0.72
1800	2.42	0.47
2000	2.58	0.16
2400	2.70	0.12

From Table 4 and the load-bearing characteristics for different bolt lengths, once the bolt length reaches a certain value, any further increase of the bolt length will have only a limited effect on the reinforcement of the rock. This is because the deeper surrounding rock is stable and has a good stress state, the effect of bolt is diminished. Therefore, once the bolt length reaches a certain value, simply increasing the bolt length does not effectively improve the strength of the reinforced rock.

### The effect of bolt diameter on the load-bearing characteristics of the reinforced rock

**Fig. 9:**
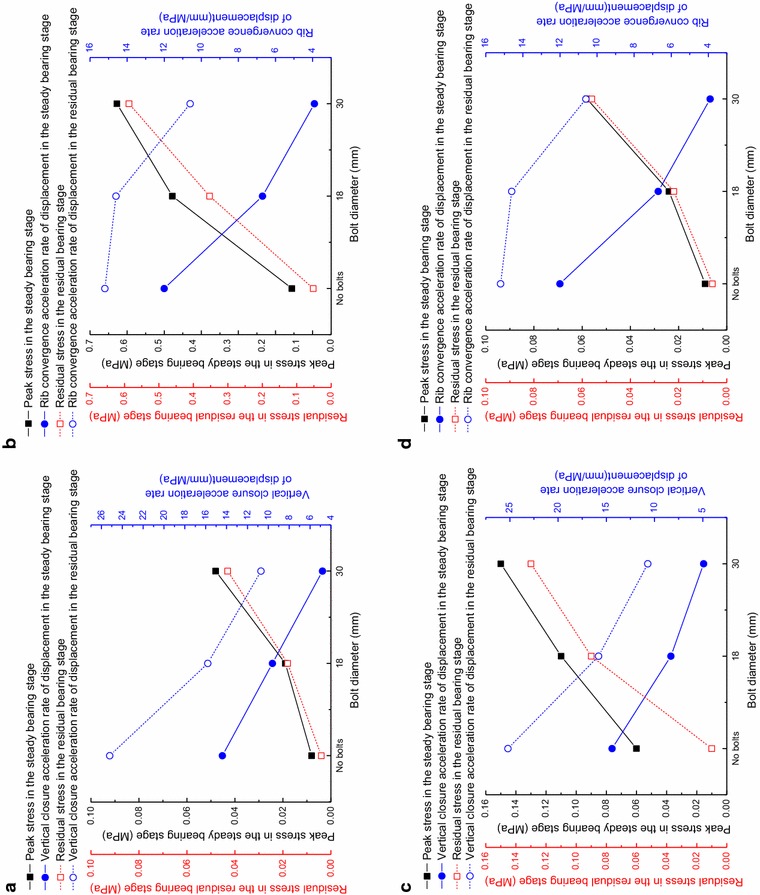
Load-bearing characteristics of the composite rock-bolt bearing structure with different bolt diameter. **a** Vertical stress at Sensor 1; **b** Vertical stress at Sensor 2; **c** Horizontal stress at Sensor 1; **d** Horizontal stress at Sensor 2

The measured stress inside the reinforced rock and the roadway deformation rate versus load for different bolt diameters are listed in Table 5. From Table 5, the load-bearing characteristics of the reinforced rock for different bolt diameters are obtained, as shown in Fig. 9.

**Table 5:** The measured stress of the composite rock-bolt bearing structure and acceleration rate of surrounding rock displacement

Bolt diameter/mm	Second stage			Third stage			Percentage of stress decrease (%)
	Kinetic pressure coefficient range	Peak stress/MPa	Vertical closure acceleration rate of displacement/mm/MPa	Kinetic pressure coefficient range	Residual stress/MPa	Vertical closure acceleration rate of displacement/mm/MPa	
*(a) Vertical stress at Sensor 1*							
No bolts	0.50–1.00	0.008	14.4	1.00–1.23	0.004	25.2	50.0
18	0.50–1.47	0.019	9.6	1.47–1.95	0.018	15.8	18.2
30	0.50–2.13	0.048	4.8	2.13–2.80	0.043	10.7	10.4

Bolt diameter/mm	Second stage			Third stage			Percentage of stress decrease (%)
	Kinetic pressure coefficient range	Peak stress/MPa	Rib convergence closure acceleration rate of displacement/mm/MPa	Kinetic pressure coefficient range	Residual stress/MPa	Rib convergence closure acceleration rate of displacement/mm/MPa	
*(b) Vertical stress at Sensor 2*							
No bolts	0.50–1.00	0.114	12.0	1.00–1.23	0.052	15.2	54.4
18	0.50–1.71	0.461	6.7	1.71–1.95	0.353	14.6	23.4
30	0.50–2.35	0.622	3.9	2.35–2.80	0.587	10.6	5.6

Bolt diameter/mm	Second stage			Third stage			Percentage of stress decrease (%)
	Kinetic pressure coefficient range	Peak stress/MPa	Vertical closure acceleration rate of displacement/mm/MPa	Kinetic pressure coefficient range	Residual stress/MPa	Vertical closure acceleration rate of displacement/mm/MPa	
*(c) Horizontal stress at Sensor 1*							
No bolts	0.50–1.00	0.060	14.4	1.00–1.23	0.012	25.2	80.0
18	0.50–1.71	0.111	9.6	1.71–1.95	0.090	15.8	18.9
30	0.50–2.13	0.148	4.8	2.13–2.80	0.128	10.7	13.5

Bolt diameter/mm	Second stage			Third stage			Percentage of stress decrease (%)
	Kinetic pressure coefficient range	Peak stress/MPa	Rib convergence closure acceleration rate of displacement/mm/MPa	Kinetic pressure coefficient range	Residual stress/MPa	Rib convergence closure acceleration rate of displacement/mm/MPa	
*(d) Horizontal stress at Sensor 2*							
No bolts	0.50–1.00	0.009	12.0	1.00–1.23	0.006	15.2	33.3
18	0.50–1.71	0.024	6.7	1.71–1.95	0.022	14.6	8.3
30	0.50–2.13	0.058	3.9	2.13–2.80	0.056	10.6	3.4

From Fig. 9, it can be seen that using large-diameter bolts in the roadway bolt support can produce larger working resistance while the same displacement is produced in the surrounding rocks. Similarly, with the same load, the displacement is much smaller when large-diameter bolts are used compared to smaller-diameter bolts. The measured data also confirm this trend. For example, the increasing rate vertical closure was 14.4 mm/MPa without support; that value decreases 33.3 % to 9.6 mm/MPa when bolts of 18 mm diameter were used, and the value decreased 66.7 % to 4.8 mm/MPa when bolts of 30 mm diameter were used. This suggests that the use of large-diameter bolts has a pronounced effect on the control of the deformation of the surrounding rock.

## Conclusion

1. There is a good correlation between the variation of the measured stress inside the reinforced rock and the variation of the surrounding rock displacement. In the early stage of the load, the stress and the surrounding rock displacement increase; in the middle stage, with the synergistic effect between the bolt and the surrounding rocks, the stress increases rapidly to the peak stress. The load-bearing ability is obtained for the reinforced rock, and the roadway displacement also increases. When the external load reaches critical values, the stress inside the reinforced rock reaches its peak load-bearing capacity and the surrounding rock is damaged, resulting in a rapid decrease of stress to the residual stress. At this stage, the deformation of the roadway drastically increases.
2. The roadway deformation rate versus load can reflect the ability of the surrounding rock to resist deformation due to external load. The variation of the roadway deformation rate versus load exhibits the following trends: it is small in the load-bearing stage before the peak stress is reached, it is smaller for higher-density bolt supports, it is smaller for longer bolt lengths, and it is smaller for larger-diameter bolts.
3. Increasing the density of the bolt support and the length and diameter of the bolt improves the load-bearing performance of the composite rock bolt/load-bearing structure, including its internal peak stress and residual stress. However, when the length of the bolts reaches certain values, a further increase of the bolt length has only a limited effect on the load-bearing capacity of the reinforced rock.
4. The bolt support improves the inner structure of the surrounding rock and the deterioration decreases as the distance between bolts decreases.
